# Exposures to information about castration and emotional trauma before puberty are associated with men’s risk of seeking genital ablation as adults

**DOI:** 10.1093/sexmed/qfad011

**Published:** 2023-04-13

**Authors:** Jame A Agapoff, Richard J Wassersug, Thomas W Johnson, Erik Wibowo

**Affiliations:** Department of Psychiatry, John A. Burns School of Medicine, University of Hawai’i at Manoa, 1356 Lusitana Street, 4th Floor, Honolulu, HI 96813, United States; Department of Cellular and Physiological Sciences, University of British Columbia, 2775 Laurel Street, Vancouver, BC V6T 1Z3, Canada; Department of Anthropology (Emeritus), California State University, Chico, P.O. Box 50. Chico, CA 95929, United States; Department of Anatomy, University of Otago, 270 Great King Street, Dunedin 9016, New Zealand

**Keywords:** castration, trauma, adverse childhood experiences

## Abstract

**Background:**

Little is known about childhood experiences, outcomes, and self-recollections of those men who were voluntarily castrated as adults.

**Aim:**

The study sought to determine how learning about castration before and after 13 years of age is associated with differential childhood experiences, outcomes, and self-recollections of those who were voluntarily castrated as adults.

**Methods:**

We designed a survey of voluntarily castrated individuals, who learned about castration before and after 13 years of age. Our survey consisted of both validated questionnaires and questions. Data were from 208 individuals. Both descriptive and quantitative statistics were performed.

**Outcomes:**

Learning about castration before 13 years of age is associated with more adverse childhood experiences (ACEs) such as being threatened with castration and other forms of emotional, physical, and sexual trauma.

**Results:**

As compared with those who learned about castration after 13 years of age, those who knew about castration earlier were more likely to have self-injured their penis (χ^2^_1_ = 5.342, *P* < 0.05), had thoughts of performing self-castration (χ^2^_1_ = 10.389, *P* < 0.01), witnessed animal castration (χ^2^_1_ = 10.023, *P* < 0.01), been threatened with castration as a child (χ^2^_1_ = 21.749, *P* < 0.001), had childhood physical trauma (χ^2^_1_ = 4.318, *P* < 0.05), had childhood emotional trauma (χ^2^_1_ = 3.939, *P* < 0.05), and had childhood sexual trauma (χ^2^_1_ = 5.862, *P* < 0.05).

**Clinical Implications:**

Mental health screening and support should be offered to any men seeking emasculating procedures in line with the World Professional Association of Transgender Health’s Standards of Care Version 8.

**Strengths and Limitations:**

This study had a large sample size and used a validated questionnaire to evaluate for ACEs. The average age of respondents was above 50 years of age, which may increase recall bias.

**Conclusion:**

Understanding how ACEs influence the age when some eunuchs first desire, pretend, and become castrated can help clinicians develop better assessments and treatment protocols for individuals with male-to-eunuch gender dysphoria, and other conditions in which emasculating medical procedures are requested.

## Introduction

There are individuals who seek emasculation procedures such as orchiectomy without any medical reason, many of whom identify as eunuchs. Eunuchs are one of the most marginalized and understudied gender minorities. Those who identify as eunuch were assigned male at birth (AMAB) but took action to eliminate many of their masculine features. This can be achieved though androgen-suppressing medications or genital ablation. Due to the misconceptions, prejudice, and social stigma faced by this group, they remain relatively invisible compared with other gender-diverse individuals.^1-3^

In recent years, there has been a greater effort to understand and characterize this group.^4-6^ These efforts have mainly focused around the Eunuch Archive (EA), an online eunuch community, where members can share testimonials and ask questions on moderated discussion forums. Many eunuch self-identifying individuals have responded to online surveys on a variety of experiences, outcomes, self-recollections, and demographics, to help researchers and clinicians better elucidate the clinical needs of this population.

Previous studies have linked adverse childhood experiences (ACEs) with a desire for genital ablation.^7-9^ Here, we define ACEs as any potentially traumatic events that occur before 18 years of age, which can impact a child or adolescents’ emotional and physical health. Some of these experiences include being threatened with castration by an adult and/or witnessing animal castration.^7^^,^^8^ In a recent study, comparing the experiences of eunuchs, aspiring eunuchs, and individuals who only fantasize about castration, a higher proportion of eunuchs reported a history of sexual and physical trauma.^8^

Not all individuals who undergo voluntary castration fit a narrow stereotype for gender identity and expression. Many present and identify as men. In one study of eunuchs, >40% of the sample identified their gender as eunuch. Smaller numbers identify as genderqueer, female, or transgender women.^9^ Consequently, because individuals with male-to-eunuch (MtE) gender identity identify with a gender other than the one assigned at birth, they could be classified as transgender.

The World Professional Organization for Transgender Health’s Standards of Care Version 8 are the first guidelines to address the needs of those with a MtE gender identity. The guidelines approach the care of would be eunuchs in the same way as other adults with gender incongruence or dysphoria. Given the high incidence of self-surgery and genital self-injury in this population, the Standards of Care Version 8 places a large emphasis on harm reduction on adults, while balancing the core principles of informed consent and authentic gender embodiment.^1^

More is known about the clinical symptoms, experiences, and outcomes of other gender-diverse youth. For example, research shows that gender dysphoria typically presents bimodally, emerging in childhood or in early adolescence.^10^ Research suggests those youth face high levels of ACEs and trauma^11^^,^^12^ in proportions higher than their cisgender peers.^13^ These studies focus mainly on the experiences and outcomes of those transitioning from MtF and female-to-male (FtM).

A small number of retrospective studies have looked at the ACEs of MtE gender dysphoric youth who become eunuchs. For example, in a study of 128 men who had obtained genital ablations, 45% of those in the castrated group reported a history of childhood physical and/or sexual trauma, and > 50% reported emotional trauma.^8^ These results are in keeping with past studies that found high proportions of childhood physical or sexual abuse in those with genital ablations.^4^^,^^7^^,^^14^

The present study seeks to develop greater insights into the childhood experience of the eunuch population and those who experience MtE gender dysphoria in adolescence and childhood. To do this, we developed an online survey on EA to evaluate childhood experiences, outcomes, and self-recollections of eunuchs who first learned about castration before and after 13 years of age.

Among individuals AMAB, the onset of puberty ranges from 9 to 14 years of age. Thirteen years of age was chosen as the division point in this study because that age is thought to correlate with Tanner Stage 3 or midpuberty in adolescents AMAB.^15^ This stage is partially defined by first enlargement of the penis and expanded pubic hair growth, making the changes of puberty obvious to the boy. It precedes or marks the onset of spermarche, the development of sperm, voice changes, and other significant secondary sexual characteristics.^16^ The goal here was to differentiate, as best as possible, the childhood experience and current characteristics of those who learned about castration before and after this stage.

This study aims to answer the following questions. For eunuchs and those with emasculation interests, is learning about castration before 13 years of age associated with a higher proportion of ACEs, outcomes, and self-recollections? For example, are ACEs associated with self-performed penile injury and/or having thoughts about performing self-castration? And, is learning about castration before 13 years of age associated with a younger age of desiring castration, pretending to be castrated, and actual castration? We hypothesize that learning about castration before 13 years of age will be associated with more ACEs and an earlier age of castration desire, ideas of performing self-castration, pretending to be castrated, self-performed penile injury, and actual castration. The findings presented in this study will be helpful for healthcare professional providing care to eunuchs and individuals interested in emasculating procedures such as those with MtE gender dysphoria.

## Methods

### Participants and procedures

An online survey, built on the SurveyMonkey platform was posted on the EA website between October 2016 and June 2017. The study was approved by the Institutional Review Board of California State University, Chico, and by the EA Steering Committee. Informed consent was received from all survey participants.

The survey consisted of both validated questionnaires and questions developed specifically for the survey. Participants provided consent online and the survey took ~30 to 40 minutes to complete. No incentive was given take the survey.

In total, 1023 participants completed the questionnaire. From this group, those who did not report themselves to be voluntarily castrated regardless of method of castration were excluded. From the final sample, data from 208 individuals were analyzed.

### Measures

#### Demographics

Questions on age, relationship status, education, income, gender, partner’s age, partner’s gender, living condition (childhood or current), handedness, and sexual attraction based on the Kinsey Scale were included.^17^ Sexual attraction options were “X-Asexual or nonsexual (no interest in sexual activity with others)”; “0-Exclusively heterosexual with no homosexual attraction”; “1-Predominantly heterosexual, only incidentally homosexual”; “2-Predominantly heterosexual, but more than incidentally homosexual”; “3-Bisexual (equal heterosexual and homosexual attraction)”; “4-Predominantly homosexual, but more than incidentally heterosexual”; “5-Predominantly homosexual, only incidentally heterosexual”; and “6-Exclusively homosexual.”

#### Paraphilic attraction and body modifications

Participants were asked, “Are you attracted to males lacking testicles?”; “Are you attracted to males lacking a penis?”; “Do you feel you are, or would be, more attractive without testicles”; “Do you feel you are, or would be, more attractive without a penis?”; “Do you have any tattoos?”; and “Do you have any piercings?” The answer options for each of these were “yes” or “no.”

#### Castration desire

Castration desires were assessed using a modified version of the Zurich Xenomelia Scale.^18^ There were 2 subscales: pure castration desire (4 items) and erotic attraction to castration (3 items). Each of the statements was rated on a scale from 1 (“strongly agree”) to 6 (“strongly disagree”). The internal consistencies for these subscales in our sample were 0.499 and 0.465, respectively. Participants were also asked about their age of receiving castration.

#### Religiosity

Religiosity was assessed using the Centrality of Religiosity Scale.^19^ This scale includes 15 items that can be rated on a 5-point scale based on frequency, ranging from “never” to “very often.” A higher score indicates greater religiosity. The internal consistency in our sample was 0.969.

#### Body image

Three subscales of the Multidimensional Body-Self Relations Questionnaire were included in this study. These are the appearance evaluation (7 items), appearance orientation (12 items), and body areas satisfaction subscales (9 items).^20^ The items for appearance evaluation and appearance orientations can be rated on 5-point scales ranging from “definitely disagree” to “definitely agree.” The items for body areas satisfaction can be rated on 5-point scales ranging from “very dissatisfied” to “very satisfied.” The internal consistencies for the appearance evaluation, appearance orientation, and body areas satisfaction subscales in our sample were 0.689, 0.826, and 0.834, respectively.

#### Sexual guilt

Sexual guilt was assessed using the Revised Mosher Sex-Guilt Scale.^21^ This scale has 10 sexual-related behaviors where participants stated the extent to which they agreed with each statement, with a higher score showing higher sexual guilt. The internal consistency was 0.791 in our sample.

#### Psychological well-being

Anxiety symptoms were measured with the Generalized Anxiety Disorder Screener.^22^ This scale has 7 items, which can be rated based on frequency in the last 2 weeks, ranging from 0 (“not at all”) to 3 (“nearly every day”). A higher score indicates more severe anxiety symptoms. The internal consistency in our sample was 0.917.

Depressive symptoms were measured using the Center for Epidemiologic Studies Depression Scale.^23^ The scale consists of 20 items, which can be rated based on frequency, ranging from 0 (“rarely or none of the time, less than 1 day”) to 3 (“most or all of the time, 5-7 days”). A higher score indicates more severe depressive symptoms. The internal consistency in our sample was 0.899.

#### Past experiences related to genital injury or ablation

Questions related to genital injury and ablation were the following: “Did anyone ever physically damage or injure your genitals when you were young?”; “Did anyone ever threaten to castrate you when you were young?, “Have you ever tried damaging or removing your penis?”; “Have you ever thought of castrating yourself?”; “When you were young, did you ever observe or assist in the castration of animals?”; and “Did you ever ‘play’ at castration? (e.g. concealing or reducing the appearance of genitals through tucking, or banding your scrotum, etc.)”. The answer options for these questions were “yes” and “no.” In addition, participants were asked to report their age of first performing self-penile injury, being threatened with castration, receiving genital injury inflicted by others, pretending to be castrated, and witnessing animal castration.

Experiences of physical, emotional and sexual traumas were also assessed using the Childhood Trauma Questionnaire.^24^ The questionnaire consists of 12 items measuring participants’ experience with physical, emotional and sexual trauma with 4 items in each category. Each can be rated from “never” to “very often.” A higher score indicates more frequent trauma. Scores above zero for each subscale indicate the presence of childhood trauma. The internal consistencies for the physical, emotional, and sexual trauma subscales in our sample were 0.83, 0.90, and 0.91, respectively.

#### Childhood toy play

Questions about our participants’ history of childhood play with male toy action figures that lacked genitalia, such as the Ken and GI Joe dolls, were assessed. Participants were asked, “How often, when you were a child, did you play with a Ken doll, a GI Joe, or some other male doll that did not have genitals?” with the following responses: “0 Never,” “1 Occasionally (few times a year),” “2 Seldom (few times a month),” “3 Often (1-2 times per week),” “4 Frequently (more than 2 times per week).” These responses were converted to binary “yes” (occasionally to frequently) or “no” (never) responses.

### Data analyses

SPSS (version 26; IBM Corporation) was used for all statistical analyses. Descriptive statistics were used to summarize various parameters. Demographic data were compared between those who learned about castration before (n = 82) and after (n = 125) 13 years of age using either *t* test for continuous variables or chi-square test for categorical variables. This categorization was based on the response to the question, “At about what age did you first learn about castration?” in which participants responded by entering a number for their age. For the regressions, those who learned about castration before or after 13 years of age were coded as “2” and “1,” respectively. Logistic regression analyses were performed to determine how childhood experiences (including exposure to castration before 13 years of age) were associated with self-performed penile injury or having thoughts of performing self-castration, while adjusting for childhood living condition and handedness. The dependent variables for the logistic regressions were past experience of self-penile injury or self-castration ideation. Linear regression analyses were performed to indicate how childhood experiences (including exposure to castration before 13 years of age) were associated with age of castration, age when castration desire first appeared, and age when they first pretended to be castrated, while adjusting for childhood living condition and handedness. The dependent variables for the linear regressions were age of castration, age when castration desire first appeared, and age when they first pretended to be castrated. The independent variables for the regressions were childhood experiences of witnessing animal castration, being threatened with castration, trauma (physical, emotional, sexual), the age when they learned about castration, childhood living condition, and handedness. All variables were included in the regression analyses using the “enter” method. *P* values <.05 were considered statistically significant.

**Table 1:** Demographic characteristics of voluntarily castrated individuals who learned about castration before and after 13 years of age.

	Before age 13 y	After age 13 y	*P* value
Castration method			.712
Chemically castrated	15 (18.3)	21 (16.8)	
Nullified	9 (11.0)	9 (7.2)	
Physically castrated	41 (50.0)	63 (50.4)	
Surgically castrated	17 (20.7)	32 (25.6)	
Age	51.5 (16.1)	52.1 (16.0)	.793
On androgen therapy	31 (37.8)	48 (38.4)	.931
Self-identified gender			.499
Male	33 (40.2)	40 (32.3)	
Eunuch	31 (37.8)	54 (43.5)	
Other gender	18 (22.0)	30 (24.2)	
In a relationship	50 (61.0)	80 (64.0)	.660
Partner’s gender for those in a relationship			.495
Female	42 (85.7)	62 (77.5)	
Male	5 (10.2)	14 (17.5)	
Others	2 (4.1)	4 (5.0)	
Partner’s age	51.1 ± 14.4	52.4 ± 14.9	.660
Childhood living condition			.012
Nonrural	51 (62.2)	97 (78.2)	
Rural	31 (37.8)	27 (21.8)	
Current living condition			.244
Nonrural	59 (72.8)	99 (79.8)	
Rural	22 (27.2)	25 (20.2)	
Highest education attainment			.061
Had university education	35 (42.7)	70 (56.0)	
No university education	47 (57.3)	55 (44.0)	
Income			.098
Under $60 000	51 (64.6)	66 (52.8)	
Above $60 000	28 (35.4)	59 (47.2)	
Religion			.889
Christian	40 (48.8)	59 (47.2)	
Other religion	7 (8.5)	9 (7.2)	
Nonreligious	35 (42.7)	57 (45.6)	
Kinsey Scale for Sexual Attraction			.802
0	16 (19.8)	17 (13.6)	
1	9 (11.1)	21 (16.8)	
2	7 (8.6)	7 (5.6)	
3	10 (12.3)	19 (15.2)	
4	4 (4.9)	9 (7.2)	
5	7 (8.6)	10 (8.0)	
6	12 (14.8)	16 (12.8)	
X^a^	16 (19.8)	26 (20.8)	
Handedness			.038
Right-handed	71 (86.6)	93 (75.0)	
Ambidextrous	8 (9.8)	13 (10.5)	
Left-handed	3 (3.7)	18 (14.5)	

Values are n (%) or mean ± SD.

a Asexual.

## Results

### Demographics

A majority of demographic variables were similar between participants who learned about castration before or after 13 years of age (Table 1). The only exceptions were childhood living condition and handedness information, with those who learned about castration younger being more likely to grow up in rural settings (37.8% vs 21.8%; χ^2^_1_ = 6.271, *P* < 0.05) and be non–right-handed (86.6% vs 75.0%; χ^2^_1_ = 6.566, *P* < 0.05).

### Childhood experiences

As compared with those who learned about castration after 13 years of age (Table 2), those who knew about castration earlier were more likely to have self-injured their penis (χ^2^_1_ = 5.342, *P* < 0.05), had thoughts of performing self-castration (χ^2^_1_ = 10.389, *P* < 0.01), witnessed animal castration (χ^2^_1_ = 10.023, *P* < 0.01), been threatened with castration as a child (χ^2^_1_ = 21.749, *P* < 0.001), had childhood physical trauma (χ^2^_1_ = 4.318, *P* < 0.05), had childhood emotional trauma (χ^2^_1_ = 3.939, *P* < 0.05), and had childhood sexual trauma (χ^2^_1_ = 5.862, *P* < 0.05). The proportion of those who had genital injuries inflicted by others, played with male action figures without genitals, and had pretended to be castrated were similar regardless of age of castration exposure.

Additionally, those who learned about castration before 13 years of age reported to have younger age of actual castration (*t*_149_ = −2.353, *P* < 0.05), first desiring castration (*t*_198_ = −5.477, *P* < 0.001), first pretending to be castrated (*t*_146_ = −3.117, *P* < 0.01), and first witnessing animal castration (*t*_60_ = −3.390, *P* < 0.001) than those who learned about castration at an older age. Ages of first masturbation, self-penile injury, being threatened with castration, and having genital injury inflicted by others were comparable regardless of age of exposure to castration.

Similarly, other outcome variables as adults were comparable regardless of when they first learned about castration, including paraphilic attraction to men without genitals, feeling attractive without genitals, having body modifications (tattoos or piercing), religiosity, sexual guilt, body image, anxiety symptoms, depressive symptoms, and levels of current castration desire.

**Table 2:** Comparison for various variables between those who learned about castration before and after 13 years of age.

Variable	Time when first learned about castration		*P* value
	Before age 13 y	After age 13 y	
Childhood experiences			
Ever self-injured penis	35 (42.7)	34 (27.2)	.021
Had self-castration thoughts	62 (84.9)	74 (63.2)	.001
Ever witnessed animal castration	35 (42.7)	27 (22.0)	.002
Ever threatened by castration	23 (28.0)	6 (4.9)	<.001
Had physical trauma	41 (50.6)	45 (36.0)	.038
Had emotional trauma	50 (61.7)	59 (47.6)	.047
Had sexual trauma	37 (46.3)	37 (29.6)	.015
Ever had genital injuries inflicted by others	10 (12.3)	13 (10.5)	.680
Ever pretended to be castrated (eg, tucking testicles between legs)	62 (75.6)	91 (72.8)	.653
Self-recollection for			
Age of castration, y	43.7 ± 15.1	49.4 ± 14.3	.020
Age of first castration desire, y	19.1 ± 15.4	31.2 ± 14.9	<.001
Age of pretending to be castrated, y	14.4 ± 8.9	20.7 ± 13.5	.002
Age of witnessing animal castration, y	8.9 ± 4.2	13.6 ± 6.5	<.001

Values are n (%) or mean ± SD.

### Age of castration exposure

Table 3 shows that exposure to castration before 13 years of age significantly increased the odds of having self-castration ideation (odds ratio, 4.076; 95% confidence interval, 1.623–10.239) but not for performing self-penile injury. Table 4 indicates that exposure to castration before 13 years of age was significantly associated with earlier age of desiring castration (β = −0.312, *P* < 0.001) but not for age of castration or age when they first pretended to be castrated. Childhood emotional trauma was associated with younger age of castration (β = −0.180, *P* < 0.05) or younger age when they first pretended to be castrated (β = −0.206, *P* < 0.05). Being threatened with castration as a child was associated with younger age of castration (β = −0.221, *P* < 0.05) and younger age when they first wanted to be castrated (β = −0.164, *P* < 0.05).

**Table 3:** Logistic regressions indicating how childhood experiences are associated with self-performed penile injury and having idea of performing self-castration, after adjusting for childhood living condition and handedness.

**Variable**	**Odds ratio**	**95% confidence interval**	***P* value**
Self-penile injury			
Ever witnessed animal castration	1.252	0.585–2.678	.563
Ever threatened by castration	1.213	0.474–3.106	.687
Had physical trauma	0.864	0.420–1.778	.692
Had emotional trauma	1.464	0.728–2.943	.285
Had sexual trauma	1.895	0.963–3.727	.064
Learning about castration before age 13 y^a^	1.370	0.696–2.698	.362
Had thought of performing self-castration			
Ever witnessed animal castration	1.097	0.461–2.609	1.097
Ever threatened by castration	0.410	0.123–1.369	.410
Had physical trauma	1.270	0.574–2.809	1.270
Had emotional trauma	0.765	0.358–1.633	.765
Had sexual trauma	1.290	0.582–2.860	1.290
Learning about castration before age 13 y^a^	4.076	1.623–10.239	4.076

a Learning about castration before 13 years old were coded as 1 for “no” and 2 for “yes.”

**Table 4:** Linear regressions showing how childhood experiences are associated to the age of castration, age when castration desire first appeared, and age when first pretended to be castrated, after adjusting for childhood living condition and handedness.

Variable	*B*	SE	β	*P* value	*R* ^2^	*F*	Model *P* value
Age of castration					0.160	F(8,138) = 3.292	.002
Ever witnessed animal castration	4.778	3.010	0.149	.115			
Ever threatened by castration	−9.349	3.764	−0.221	.014			
Had physical trauma	0.341	2.882	0.011	.906			
Had emotional trauma	−5.324	2.676	−0.180	.049			
Had sexual trauma	−0.213	2.723	−0.007	.938			
Learning about castration before age 13 y^a^	−5.195	2.773	−0.171	.063			
Age of first castration desire					0.230	F(8,183) = 6.827	<.001
Ever witnessed animal castration	4.835	2.648	0.136	.070			
Ever threatened by castration	−8.485	3.607	−0.164	.020			
Had physical trauma	−3.050	2.544	−0.093	.232			
Had emotional trauma	−4.287	2.443	−0.132	.081			
Had sexual trauma	−0.397	2.417	−0.012	.870			
Learning about castration before age 13 y^a^	−10.393	2.421	−0.312	<.001			
Age of first pretending to be castrated					0.124	F(8,132) = 2.334	.022
Ever witnessed animal castration	0.820	2.499	0.031	.743			
Ever threatened by castration	−0.783	3.094	−0.023	.801			
Had physical trauma	−0.755	2.320	−0.030	.745			
Had emotional trauma	−5.048	2.259	−0.206	.027			
Had sexual trauma	−1.650	2.282	−0.065	.471			
Learning about castration before age 13 y^a^	−4.594	2.347	−0.184	.052			

a Learning about castration before 13 years old were coded as 1 for “no” and 2 for “yes.”

## Discussion

In this study, we aimed to better understand how learning about castration before or after 13 years of age influences interest in castration for men who subsequently elected voluntary castration (ie, the MtE population). We also wanted to better understand how childhood experiences—particularly those of a potential traumatic nature—influenced subsequent genital self-injury and ablation for this population. This age is thought to correlate with Tanner Stage 3 or midpuberty in individuals AMAB.^15^ Tanner Stage 3 precedes spermarche and the emergence of significant secondary sexual characteristics,^16^ plus age-appropriate sexual activities, such as masturbation. The changes associated with puberty would become obvious to a boy at this stage and might be distressing for those who experience MtE gender dysphoria. Our results demonstrate some significant similarities and differences between the groups similar to those observed in those with MtF and FtM gender dysphoria.^25^

Demographically, our groups only differed in childhood living condition and handedness, with a majority of the respondents, who learned about castration before 13 years of age, coming from rural environments. A larger proportion of this group also reported witnessing animal castration at a younger age, which would make sense if they lived on or near a farm.

Despite their disparate childhood environments, a majority would later settle in nonrural environments, perhaps due the perceived prejudice of rural communities, become partnered, and engage predominantly in heterosexual relationships. A majority of both groups described being attracted to men without testicles and felt attractive without testicles, indicating possible bisexual tendencies.

Previous research has found that many eunuchs come from a strongly religious Christian upbringing.^4^^,^^5^^,^^7^ The results of this analysis showed low levels of religiosity and sexual guilt. Nearly half of our sample reported being of Christian faith, but <5% described religiosity or sexual shame. Low religiosity in this sample may represent a rejection of past negative religious experiences. Sexual guilt is often associated with conservative religious beliefs that question the morality of sexual expression; therefore, it is reasonable that a group with low religiosity would also have little sexual shame.

As shown in Table 2, there were significant differences in childhood experiences and self-recollections between the groups. For example, individuals who learned about castration before 13 years of age were more likely to have experienced sexual, emotional, or physical trauma, or to have been threatened with castration.

Gender-diverse children are known to experience high levels of adverse experiences and trauma.^11^^,^^12^ In one cross-sectional study of transgender adults who completed the Childhood Trauma Questionnaire, over 30% reported severe to extreme childhood aversities, and 25% reported violent behaviors by parents.^26^ Higher proportions of childhood trauma were also found in our sample, with 46.3% to 61.7% of those who learned about castration before 13 years of age reporting a trauma history compared with 29.6% to 47.6% of those who learned about castration after 13 years of age. In the Biedermann et al sample,^26^ nearly 70% experienced childhood bullying. Similar to other gender-diverse youth, individuals who eventually seek emasculation may be targeted for harassment and aggression because their behavior does not fall in line with typical male gender norms. This makes early interventions critical to support individuals, who show an interest in genital ablation or have a history of genital self-injury, without a desire for feminization, as critical as it is for individuals who fit a more conventional MtF profile.

In our sample, a higher proportion of individuals who learned about castration before 13 years of age had self-castration thoughts, and reported having self-injured their own penis. This population, as a whole, has a high rate of genital self-injury.^9^^,^^27^ There are several possible explanations for this. Individuals who injure their genitals may see it as the only means to access proper medical treatment and care such as an orchiectomy.^5^^,^^28^ Until September 2022, no standard of care was available for would-be eunuchs,^1^ which made access to care through traditional medical avenues unreliable at best.

Self-injury may also be associated with mental health pathologies like depression and posttraumatic stress disorder, which occur at higher rates in transgender youth and other marginalized persons.^29-31^ For example, in the 2015 U.S. Transgender Survey, 53% of youth surveyed reported serious psychological distress in the prior month.^30^ Access to appropriate and timely gender-affirming care is known to significantly improve the health and well-being of gender-diverse youth.^1^^,^^30^ Given the low depression and anxiety scores in our adult eunuch sample, MtE youth would also benefit from gender-affirmative support.

In our sample, eunuchs who learned about castration before 13 years of age more often pretended to be castrated at an earlier age, reported an earlier age of first desiring castration, and reported an earlier age of castration (Table 2). We do not believe this finding to be coincidental. Gender-diverse children are known to experience high levels of adverse experiences and trauma.^11^^,^^12^ As stated previously, nearly half of all eunuchs who heard about castration before 13 years of age reported physical, emotional, or sexual trauma. This high occurrence of exposure to trauma is also observed in individuals who are interested in or considering castration.^8^

Of note, eunuchs who learned about castration before 13 years of age were more likely to have witnessed animal castration (42.7%) and to have observed it at an earlier age (8.9 vs 13.6 years of age) (ie, before reaching Tanner Stage 3) (Table 2). This early exposure to animal castration has been observed in other studies of eunuchs.^8^^,^^9^ Witnessing animal castration may be when many eunuchs first learn about castration. That nearly half of our eunuch sample who learned about castration before 13 years of age witnessed animal castration seems significant and may relate to the fact that close to 40% of them grew up in rural settings. It is possible that seeing this procedure, both its apparent simplicity and its perceived positive effects, may motivate some already experiencing gender dysphoria to seek future emasculation. It may also be a predictor of future attainment of becoming a eunuch.

Interestingly, eunuchs who learned about castration before and after 13 years of age did not differ in current characteristics. It may be that individuals who transition from male to eunuch experience the onset of their gender dysphoria at different time points, similar to those on the MtF/FtM spectrum, but ultimately they experience the same outcomes as adults.

In our sample, a majority of both groups expressed attraction to men without testicles and felt attractive without testicles. Neither group reported high levels of depression or anxiety symptoms after their castration. The absence of discrete differences in anxiety and depression symptoms between the groups could be interpreted as one beneficial outcome of achieving castration. Access to gender-affirmative care, including medical interventions, is known to reduce significant psychopathologies in transgender persons including transgender youth.^1^ In this way, individuals with MtE gender dysphoria, who receive orchiectomy, may consequently experience improvements in mood symptoms.

Table 3 shows logistic regressions indicating how childhood experiences are associated with self-performed penile injury and having thoughts about performing self-castration. The odds of self-penile injury were not increased in those having experiences of trauma, including threats of castration, witnessing animal castration, or learning about it before 13 years of age. Similarly, the odds of having thoughts of performing self-castration were not increased by physical, emotional, or sexual trauma, nor by being ever threatened with castration or observing animal castration. In fact, learning about castration after 13 years of age seemed to convey a protective effect, with there being decreased odds of having thoughts of performing self-castration. These results collectively emphasize that it is not trauma or other childhood experiences that drive self-penile injury or self-castration thoughts, but rather a desperate attempt to correct MtE gender dysphoria in the absence of safe and affirming medical interventions.

Table 4 outlines linear regressions showing how childhood experiences are associated with the age of castration, the age when castration desire first appeared, and the age when one first pretended to be castrated. After adjusting for childhood living conditions and handedness, experiencing emotional trauma was inversely proportional to the age of first pretending to be castrated and with castration itself. Being threatened with castration trended similarly, showing significant inverse relationships with the age of first castration desire and actual castration. Learning about castration before 13 years of age was associated with younger onset of castration desire.

These correlations emphasize the complex interplay between MtE gender dysphoria, trauma, and age-related castration outcomes. Emotional trauma and being threatened with castration appear particularly important, as they influence more than 1 dependent variable. One explanation for these findings is that particular types of negative exposures uncover MtE gender dysphoria at a younger age, leading to earlier castration desire, pretend play, and ultimately castration. As with other results presented in this study, the presence of trauma and ACEs are not thought to have a causal role in MtE gender dysphoria, but rather are thought to have an influence on how that dysphoria is experienced and acted upon by those who become eunuchs.

## Limitations

This study has several limitations. All data were collected online. We could not validate the accuracy or biases in some responses (eg, whether or not individuals first learned about castration before or after 13 years of age). The average age of respondents was above 50 years of age, which may increase recall bias. Because participants were recruited from a website dedicated to castration topics, our data may only be generalizable to individuals with elevated interest in genital ablation.

## Conclusion

Our results help to elucidate the unique characteristics of the MtE population, and how a knowledge of castration is associated with differential experiences, outcomes, and self-reflections depending on the age of exposure. The presence of trauma and ACEs does not play a causal role in MtE gender dysphoria, but rather it alters the experiencing of that dysphoria. Trauma or other childhood experiences do not drive self-penile injury or self-castration thoughts. However, learning about castration before 13 years of age may determine the mean age of castration desire. As other factors, like desperate attempts to correct MtE gender dysphoria in the absence of safe and affirming medical interventions, may drive self-penile injury or self-castration thoughts, all MtE individuals should be under medical supervision.
